# Smad3-related miRNAs regulated oncogenic TRIB2 promoter activity to effectively suppress lung adenocarcinoma growth

**DOI:** 10.1038/cddis.2016.432

**Published:** 2016-12-22

**Authors:** Yan-Xia Zhang, Yun-Fei Yan, Yue-Mei Liu, You-Jie Li, Han-Han Zhang, Min Pang, Jin-Xia Hu, Wei Zhao, Ning Xie, Ling Zhou, Ping-Yu Wang, Shu-Yang Xie

**Affiliations:** 1 Key Laboratory of Tumor Molecular Biology in Binzhou Medical University, Department of Biochemistry and Molecular Biology, Binzhou Medical University, YanTai, ShanDong, P.R.China; 2 Department of Chest Surgery, YanTaiShan Hospital, YanTai, ShanDong, P.R.China

## Abstract

MicroRNAs (miRNAs) and Smad3, as key transcription factors in transforming growth factor-*β*1 (TGF-*β*1) signaling, help regulate various physiological and pathological processes. We investigated the roles of Smad3-regulated miRNAs with respect to lung adenocarcinoma cell apoptosis, proliferation, and metastasis. We observed that Smad3 and phospho-SMAD3 (p-Smad3) were decreased in miR-206- (or miR-140)-treated cells and there might be a feedback loop between miR-206 (or miR-140) and TGF-*β*1 expression. Smad3-related miRNAs affected tribbles homolog 2 (TRIB2) expression by regulating trib2 promoter activity through the CAGACA box. MiR-206 and miR-140 inhibited lung adenocarcinoma cell proliferation *in vitro* and *in vivo* by suppressing p-Smad3/Smad3 and TRIB2. Moreover, lung adenocarcinoma data supported a suppressive role for miR-206/miR-140 and an oncogenic role for TRIB2—patients with higher TRIB2 levels had poorer survival. In summary, miR-206 and miR-140, as tumor suppressors, induced lung adenocarcinoma cell death and inhibited cell proliferation by modifying oncogenic TRIB2 promoter activity through p-Smad3. MiR-206 and miR-140 also suppressed lung adenocarcinoma cell metastasis *in vitro* and *in vivo* by regulating EMT-related factors.

Lung cancer causes more than 1 600 000 new lung cancers each year^1^ and contributes to greater than 1 370 000 cancer-related deaths^2^ worldwide, making it the most fatal of all cancers. Of all lung cancers,^3^ 85% are non-small-cell lung cancers^4, 5^ and adenocarcinomas are the most prevalent. Although non-small-cell lung cancers can be diagnosed early, they are often diagnosed late, when prognosis is poor.^4^ Over the past 30 years, overall 5-year lung cancer survival is ~16%.^3^ Therefore, new molecularly targeted therapy is urgently needed.

Transcription factor Smad3 is a central downstream modulator of transforming growth factor-*β* (TGF-*β*1)/Smad signaling, participating in the regulation of various physiological and pathological processes, including carcinogenesis.^6^ Cancer cell metastasis is a chief cause of lung cancer mortality. Smad3 is a central signaling molecule of TGF-*β*1, inducing epithelial-to-mesenchymal transition (EMT), during which early stage tumors are converted into invasive malignancies.^7^ Overexpression of Smad3 promoted metastasis in mice injected with human metastatic breast cancer cells (MCF10CA1a), but a COOH-terminally truncated dominant negative mutant of Smad3 suppressed cell metastasis.^8^

MiRNAs are noncoding RNAs of 20–22 nucleotides that bind to the 3′-untranslated regions (3′-UTRs) of cognate mRNAs, negatively regulating target mRNAs.^9, 10^ miRNAs, as oncogenes or tumor suppressive genes, have been reported to modulate cell growth, metastasis, and cell death.^11^ MiR-206^12^ and miR-140^13^ act as tumor suppressive genes in the tumorigenesis. MiR-16 has also been verified to act as a tumor suppressor by downregulating BCL-2, whereas miR-150, by negatively regulating p53 expression, was confirmed to be an oncogene.^14, 15^ MiR-27a can function as an oncogene by targeting MAP2K4, and inhibition of miR-27a increases MAP2K4 expression, which subsequently inhibits MG63 cell proliferation and migration.^16^

Considering potential roles of miRNAs and Smad3 in tumor cell growth and metastasis, we studied functions of Smad3-related miRNAs in lung cancer cell apoptosis, proliferation, and metastasis, and confirmed that miR-206 and miR-140 can suppress tumors as well as regulate phospho-Smad3 (p-Smad3)/Smad3, which can affect TRIB2 and suppress lung adenocarcinoma cell proliferation or metastasis.

## Results

### miR-206 and miR-140 inhibited lung adenocarcinoma cell proliferation

**Figure 1:**
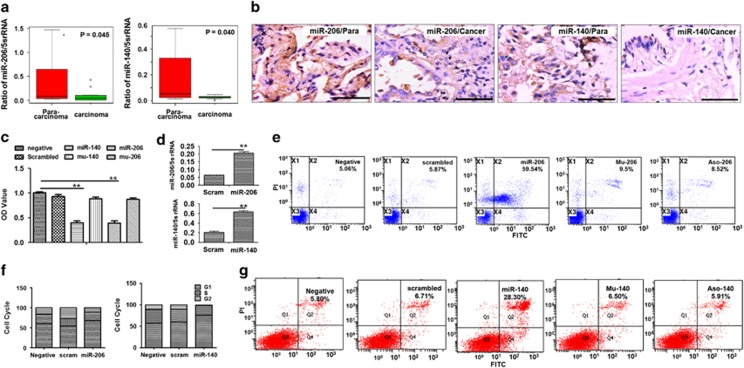
miR-206 and miR-140 regulate A549 cell growth. (**a**) Real-time PCR. Relative fold changes of miR-206 (or miR-140) were decreased in lung adenocarcinoma samples (*n*=10) compared with para-carcinomas (*n*=10). (**b**) *In situ* hybridization detection of miR-206 or miR-140. The brown color in cell indicates the expression of miRNAs. Bar=50 *μ*m. (**c**) A549 cell growth according to MTT assay. OD was less in miR-206- (*P*<0.01) or miR-140- treated A549 cells (*P*<0.01) compared with negative or control mutant-oligo-treated cultures. (**d**) miRNAs measured with real-time PCR. miR-206 (or miR-140) were higher in miR-206- (or miR-140)-treated cells compared with scrambled-oligo controls. **P<0.01. miR-206 (or miR-140) treatment *versus* control treatment. (**e**) FACS analysis of miR-206-induced apoptosis. (**f**) Cell cycle distribution of A549 cells transfected with miR-206 and miR-140. All experiments were carried out in triplicate. A significant increase in A549 cells in the G1 phase occurred in miR-206- or miR-140-treated cells compared with control cultures. (**g**) FACS analysis of miR-140-induced apoptosis. Apoptotic cells are shown in the upper left and right, and lower right quadrants of each panel. Apoptotic cells were increased in miR-206- or miR-140-treated cells compared with scrambled- or mutant mimics (Mu-206 or Mu-140) -treated cells after Annexin V-FITC/PI staining. Negative, vehicle-treated cells without oligos. Scrambled (scram), scrambled oligo control RNA. MiR-206 or miR-140, cells treated with miR-206 or miR-140 oligos. Mu-206 or Mu-140, cells treated with mutation sequence of miR-206 or miR-140. ASO-206 or ASO-140, cells treated with antisense RNA specific to miR-206 or miR-140

miRNAs act as tumor suppressive genes or oncogenes during tumor formation, and miR-206^12^ and miR-140^13^ have been reported to be tumor suppressive genes. To further investigate their roles in lung adenocarcinoma, we measured miR-206 and miR-140 expression in lung adenocarcinoma samples, and noted that miR-206 and miR-140 expression decreased in adenocarcinoma samples compared with para-carcinomas (*n*=10, Figure 1a). *In situ* hybridization further proved that the expression of miR-206 and miR-140 significantly decreased in type 2 epithelial cells in lung adenocarcinoma samples compared with those in para-carcinomas (Figure 1b). Next, we verified their roles in lung adenocarcinoma *in vitro*. We observed that miR-206 can inhibit lung adenocarcinoma cell (A549) proliferation (Figure 1c and d) and FACS analysis confirmed that miR-206 treatment induced more apoptosis compared with NC-oligo control, whereas the inhibition of proliferation by miR-206 was abolished with Mu-206-control treatment (Figure 1e). The role of miR-206 in inducing A549 apoptosis was abolished after application of a miR-206 inhibitor (Aso-206, Figure 1e), supporting a tumor suppressive role of miR-206. Our results revealed that A549 cells in the G1 phase increased significantly after miR-206 treatment compared with control (oligo-treated) cultures (Figure 1f), suggesting that the suppressive role of miR-206 is relevant to regulating the cell cycle, most likely due to G1 phase inhibition.

Moreover, miR-140 significantly suppressed A549 cell proliferation and induced A549 apoptosis (Figure 1c, d, and g) compared with NC- or Mu-140 control treatment. The tumor suppressive role of miR-140 with respect to cell cycle regulation is also relevant to G1 phase inhibition (Figure 1f). Similar data were confirmed in a miR-206- or miR-140-transfected lung adenocarcinoma cell line (LTEP-a-2 cells) (Supplementary Figure 1). The 3-(4,5-dimethylthiazol-2-yl)-2,5-diphenyltetrazolium bromide (MTT) assay and colony formation assay further showed that downregulation of miR-206 or miR-140 by miRNA inhibitors (ASO-206 or ASO-140) promoted cell proliferation and increased colony formation capacity of A549 cells compared with scrambled control treatment (Supplementary Figure 1), supporting miR-140 and miR-206 as tumor suppressive genes.

### Smad3 is a direct target of miR-206 and miR-140

**Figure 2:**
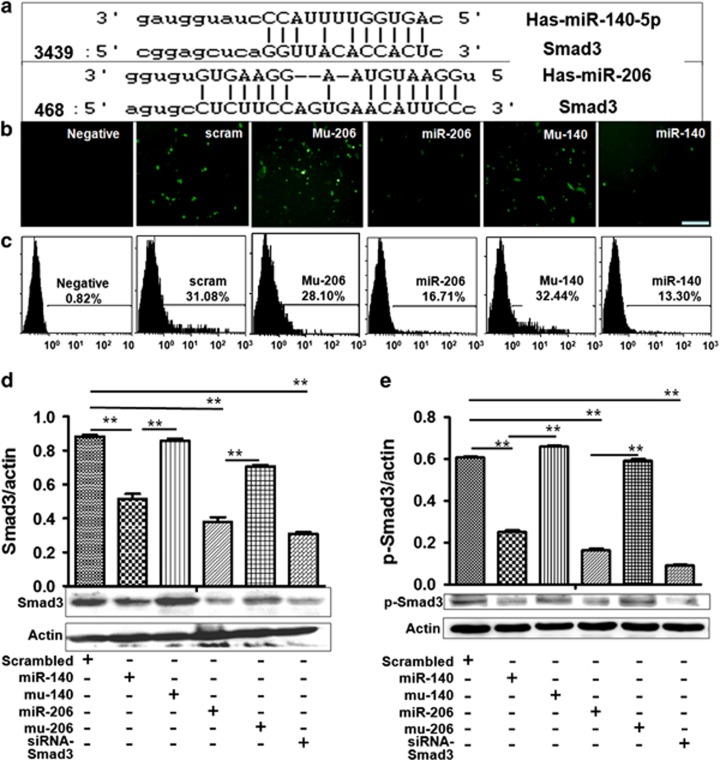
Smad3 expression is regulated by miR-206 and miR-140 in A549 cells. (**a**) The site of Smad3 3′-UTR is targeted by miR-206 and miR-140. (**b**) Fluorescent analysis (bar=100 *μ*M). (**c**) FACS analysis. GFP-positive cells and GFP fluorescent intensity in miR-206- and miR-140-treated cultures were decreased significantly compared with control culture. (**d** and **e**) Smad3 and p-Smad3 expression. Data showed that p-Smad3 and Smad3 expression decreased in miR-206 and miR-140-treated cells compared with controls. Smad3/actin (or p-Smad3/actin) is shown in the upper panel. **P<0.01, miR-206 (or miR-140) treatment *versus* scrambled or mutant Mu-206 (or Mu-140) control. *P*<0.01, siRNA treatment *versus* control. Negative, mock transfections. Scrambled, cells treated with scrambled oligo control RNA. MiR-206 or miR-140, cells treated with miR-206 or miR-140 oligos. Mu-206 or Mu-140, cells treated with mutation sequence of miR-206 or miR-140. SiRNA-smad3, small interfering RNA specific for knocking down Smad3 expression

We confirmed that the 3′-UTR of Smad3 contains the predicted target site (wild type) of miR-206 and miR-140 according to online miRNA analysis software (http://www.microrna.org/microrna/getMirnaForm.do, or http://www.targetscan.org/index.html. Figure 2a). Then, a pcDNA-GFP-smad-UTR vector was cloned with human Smad3 3′-UTR, which was transfected with miR-206 (or miR-140) into A549 cells. GFP expression was significantly decreased in miR-206- (or miR-140)-treated cells compared with controls (Figure 2b). FACS results revealed fewer GFP-positive cells in miR-206- and miR-140- treated cultures compared with NC-treated cultures (Figure 2c).

Western blot confirmed that not only Smad3 expression decreased, but also p-Smad3 levels were downregulated in miR-206- (or miR-140)-transfected cells compared with control treatment (Figure 2d and e). Similar data were obtained in miR-206- and miR-140-transfected LTEP-a-2 cells compared with control oligo treatment (Supplementary Figure 2a–c), suggesting that Smad3 is a direct target of miR-206 (or miR-140).

### Negative roles of miR-206 and miR-140 in the TGF-*β*1 pathway

**Figure 3:**
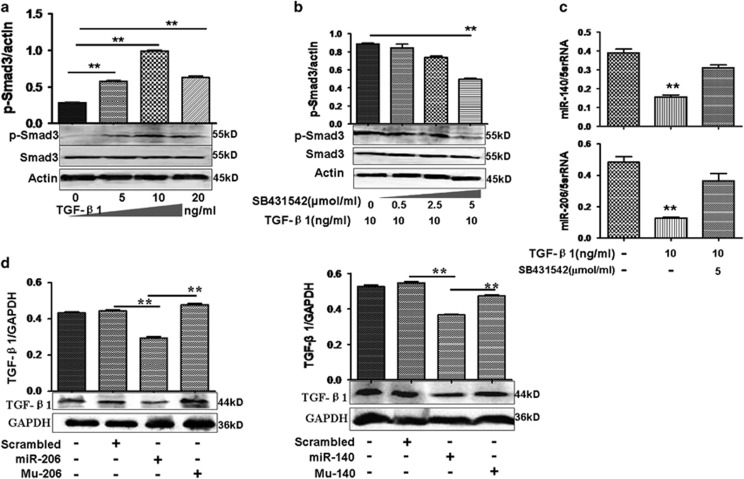
Interaction between TGF-*β*1 and miR-206/miR-140. (**a**) Effect of TGF-*β*1 (0–20 ng/ml) on p-Smad3 expression at 24 h. TGF-*β*1 (10 ng/ml) increased p-Smad3 in A549 cells. **P<0.01, 5, 10, and 20 ng/ml TGF-*β*1 treatment *versus* untreatment. The ratio of p-Smad3/actin is indicated in the upper panel. (**b**) SB431542 (5 *μ*mol/ml) suppressed p-Smad3 induced by TGF-*β*1 in A549 cells. Relative value for p-Smad3/actin appears in the upper panel. **P<0.01, 5 *μ*mol/ml SB 431542 treatment *versus* TGF-*β*1. (**c**) Effect of TGF-*β*1 on miR-206 or miR-140 expression at 24 h. miR-206 and miR-140 expression decreased with TGF-*β*1 treatment compared with untreated A549 cells. **P<0.01 TGF-*β*1 treatment *versus* untreatment. (**d**) Effect of miR-206 or miR-140 on TGF-*β*1. MiR-206 and miR-140 decreased TGF-*β*1 in A549 cells compared with scrambled-control treatment. **P<0.01 miR-206 (or miR-140) treatment *versus* scrambled or mutant Mu-206 (or Mu-140). *n*=3 replicates

Because TGF-*β*1 can induce p-Smad3 expression,^17^ we treated lung adenocarcinoma cells with different concentrations of TGF-*β*1 (0–20 ng/ml). We noted that 10 ng/ml TGF-*β*1 enhanced the ratio of p-Smad3 to Smad3 obviously in A549 cells (Figure 3a), but this was inhibited gradually after cells were treated with 0.5–5 *μ*mol/ml the TGF-*β*1 inhibitor (SB431542). The greatest effects were observed with 5 *μ*mol/ml SB431542 (Figure 3b). Thus, the concentrations of 10 ng/ml TGF-*β*1 and 5 *μ*mol/ml SB431542 were used in this study.

Next, real-time PCR data indicated that miR-206 and miR-140 were decreased in 10 ng/ml TGF-*β*1-treated cultures compared with untreated A549 cells (Figure 3c) and decreased expression of miR-206 (or miR-140) by TGF-*β*1 in A549 cells was restored after 5 *μ*mol/ml SB431542 treatment. Expression of miR-206 (or miR-140) in LTEP-a-2 cells was inhibited by TGF-*β*1 (Supplementary Figure 3), indicating that the expression of miR-206 and miR-140 was regulated by TGF-*β*1.

Then we asked whether there is any relationship between miR-206 (or miR-140) and the TGF-*β*1 pathway or whether TGF-*β*1 levels are affected by miR-206 and miR-140 in turn? The results analyzed by online miRNA analysis software did not show that 3′-UTR of TGF-*β*1 was targeted by miR-206 (or miR-140), but the TGF-*β*1 expression was downregulated in miR-206- (or miR-140)-treated A549 cells compared with controls (Figure 3d), suggesting there might be a feedback loop between miR-206 (or miR-140) and TGF-*β*1 in which miR-206 (or miR-140) can decrease TGF-*β*1 levels indirectly.

### miR-206 and miR-140 regulate TRIB2 promoter activity through Smad3-binding ‘CAGACA'

**Figure 4:**
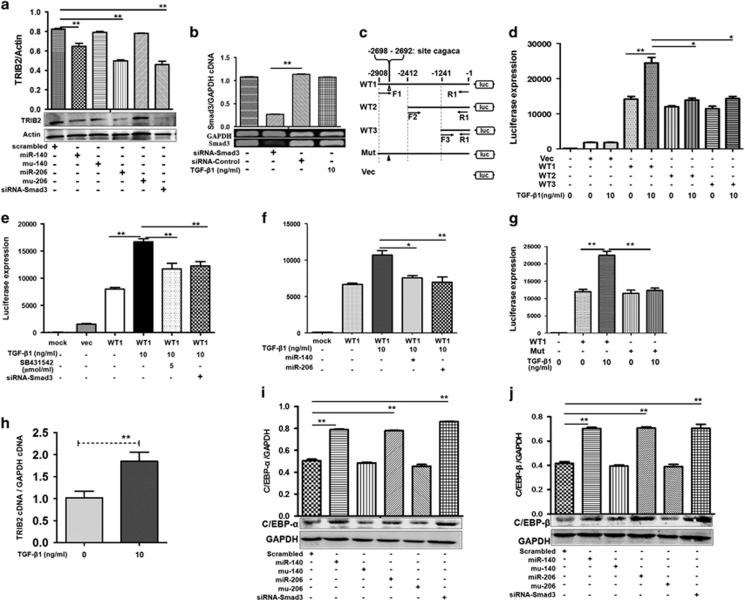
TRIB2 and its promoter activity are regulated by miR-206 and miR-140. (**a**) TRIB2 was downregulated in miR-206-, miR-140-, or siRNA-treated A549 cells. **P=0.007, miR-140 *versus* scrambled; **P<0.01, miR-206 or siRNA *versus* scrambled. Relative values for TRIB2/actin are indicated in the upper panel. (**b**) Smad3 cDNAs were reduced by siRNA treatment (specific to Smad3) compared with control oligo-treated cultures. Relative values for Smad3/GAPDH cDNAs are indicated in the upper panel. **P<0.01 *versus* siRNA control. (**c**) Sketch of TRIB2 promoter luciferase plasmids. WT1, WT2, and WT3 indicate 2.9 kb (at position −2908 to −1), 2.4 kb (at position −2412 to −1), and 1.2 kb (at position −1241 to −1) length promoters of the *TRIB2* gene (wild type), respectively. Mut, mutant TRIB2 promoter (The sites at position −2698 and −2692 was mutated). Vec, negative control plasmid. (**d**) The luciferase levels were higher in 2.9 kb length of the TRIB2 promoter (WT1) plasmid-treated cultures compared with 2.4 kb (WT2), or 1.2 kb (WT3) length promoter-treated cells, especially with TGF-*β*1 treatment at 24 h. **P<0.01 WT1 with 10 ng/ml TGF-*β*1 treatment *versus* untreatment; *P<0.05 WT2 or WT3 *versus* WT1 with TGF-*β*1 treatment. (**e**) SB431542 blocked TGF-*β*1-mediated enhancement of 2.9 kb length TRIB2 promoter activity. **P<0.01 TGF-*β*1 treatment *versus* untreatment, SB431542, or siRNA-Smad3. (**f**) miR-206 and miR-140 blocked TGF-*β*1-mediated enhancement of TRIB2 promoter activity. *P<0.05 miR-140 *versus* TGF-*β*1 treatment; **P<0.01 miR-206 *versus* TGF-*β*1 treatment. (**g**) Mutant of ‘CAGACA' box at position −2698 and −2692 of the TRIB2 promoter reduced TGF-*β*1-induced promoter activity. **P<0.01 WT1 with TGF-*β*1 treatment *versus* WT1 without TGF-*β*1, or Mut-promoter. (**h**) TGF-*β*1 increased TRIB2 transcription obviously. **P<0.01 TGF-*β*1 treatment *versus* untreatment. (**i** and **j**) Expression of C/EBP-*α* or C/EBP-*β* increased in miR-206- or miR-140-treated A549 cells compared with control treatment. **P<0.01 miR-206, or miR-140, or siRNA *versus* scrambled oligo control. Relative values for C/EBP-*α* (or C/EBP-*β*) *versus* GAPDH are indicated in the upper panel. Mock, vehicle-treated cells without a reporter plasmid. Scrambled, scrambled oligo control RNA. MiR-206 or miR-140, cells treated with miR-206 or miR-140 oligos. Mu-206 or Mu-140, cells treated with the miR-206 or miR-140 mutation sequence. SiRNA-Smad3 or SiRNA-control, cells treated with small interfering RNA specific to Smad3 or siRNA-control

Previously, a Tribble family member TRIB3 was reported to interact with Smad3.^18^ TRIB2, another Tribble family member, acts as an oncogene in acute myeloid and T-cell acute lymphoblastic leukemias^19^ and some lung cancers,^20, 21^ but whether TRIB2 and Smad3 interact is unclear. Thus, we reduced p-Smad3 expression with miR-206 or miR-140, and noted that TRIB2 was reduced in miR-206- (or miR-140)-transfected A549 cells, and the TRIB2 levels inhibited by miR-206 (or miR-140) was abolished in the mutation mimics (Mu-206 or Mu-140)-treated cultures (Figure 4a,Supplementary Figure 4), indicating that TRIB2 was also regulated by miR-206 and miR-140.

SiRNA can be used to investigate gene function and signal pathways, so we used this method to study whether miR-206 and miR-140 regulated TRIB2 expression through Smad3. Using siRNA specifically designed for Smad3, we observed that this siRNA could inhibit Smad3 mRNA compared with siRNA control-treated cells (Figure 4b). Smad3 and p-Smad3 were lower in siRNA-treated A549 cells compared with control cells, indicating that siRNA decreased Smad3 expression (Figure 2d and e, Supplementary Figure 2). To understand whether these siRNAs (siRNA-Smad3, specific to Smad3) affected the expression of TRIB2, we observed that TRIB2 expression decreased in siRNA-Smad3-treated cells compared with control treatment (Figure 4a,Supplementary Figure 4), an outcome similar to the effects of miR-206 and miR-140 on TRIB2 expression. siRNA experiments in LTEP-a-2 cells were also similar as well regarding TRIB2 expression. Therefore, Smad3-related miR-206, miR-140, and siRNA could regulate TRIB2 expression, suggesting that Smad3 may affect TRIB2 expression.

In the TGF-*β*1 pathway, p-Smad3 was reported to bind other Smad proteins together to form a protein complex, which then moves to the cell nucleus and promotes Smad3 responsive promoter activity to drive gene expression.^22^ Therefore, we studied whether Smad3 could drive *trib2* gene expression by affecting *trib2* gene promoter. Then, different lengths of TRIB2 promoter luciferase plasmids were cloned (Figure 4c) and Hela cells were treated with these plasmids. The 2.9 kb of the TRIB2 promoter (WT1) had the most activity for driving luciferase expression with TGF-*β*1 treatment for 24 h compared with the 2.4 kb (WT2) or 1.2 kb (WT3) length promoter (Figure 4d). Activity of the 2.9 kb section of the TRIB2 promoter (WT1) induced by TGF-*β*1 was blocked in cells treated with 5 *μ*mol/ml SB431542 or siRNA-Smad3 oligos (Figure 4e). We further found that miR-206 and miR-140 also effectively blocked the activity of TRIB2 promoter (WT1) induced by TGF-*β*1 (Figure 4f), which proved that TGF-*β*1/Smad3 promoted TRIB2 promoter activity. Moreover, experiments with cells treated mouse *trib2* promoter-luciferase reporter also demonstrated that TGF-*β*1 treatment enhanced mouse TRIB2 promoter activity, whereas SB431542 treatment inhibited mouse TRIB2 promoter activity induced by TGF-*β*1 for 24 h (Supplementary Figure 5), which further proved that TGF-*β*1/Smad3 could promote TRIB2 promoter activity obviously.

The activity of promoter induced by TGF-*β*1 might be related to the Smad3-binding consensus sequence CAGACA.^23^ We found that there was a sequence box ‘CAGACA' at position −2698 and −2692 of the human TRIB2 promoter and mutated this sequence to further prove whether TGF-*β*1/Smad3 promoted TRIB2 promoter activity through CAGACA. When the CAGACA box at position −2698 was mutated in the Mut-promoter plasmid using site-directed Gene Mutagenesis Kit (Supplementary Figure 6), the luciferase expression decreased in Mut-promoter plasmid-treated cultures compared with wild type (WT1) cultures. Moreover, TGF-*β*1 treatment cannot increase the luciferase levels in Mut-promoter plasmid-treated cultures (Figure 4g), which proved that Smad3 promotes TRIB2 responsive promoter activity to drive TRIB2 expression by the ‘CAGACA' box. To further investigate the role of Smad3 on TRIB2 responsive promoter activity, the transcriptional levels of TRIB2 mRNA were analyzed after TGF-*β*1 treatment. Our results showed that 10 ng/ml TGF-*β* significantly increased TRIB2 mRNA levels (Figure 4h).

The CCAAT/enhancer-binding proteins *α* and *β* (C/EBP*α* and *β*) are reported to be downstream factors of TRIB2.^24, 25^ To investigate the effects of Smad3-related miRNAs on C/EBP*α* and *β* expression, lung adenocarcinoma cells were treated with miR-140 and miR-206. Western blot revealed that expression of C/EBP*α* and *β* increased in miR-206- and miR-140-treated A549 cells compared with control treatment (Figure 4i and j). Similar results occurred in siRNA oligo-treated cultures, indicating a regulatory role for miR-206 and miR-140 with respect to TRIB2 and its downstream factors.

### miR-206 and miR-140 inhibited cell metastasis through Smad3

**Figure 5:**
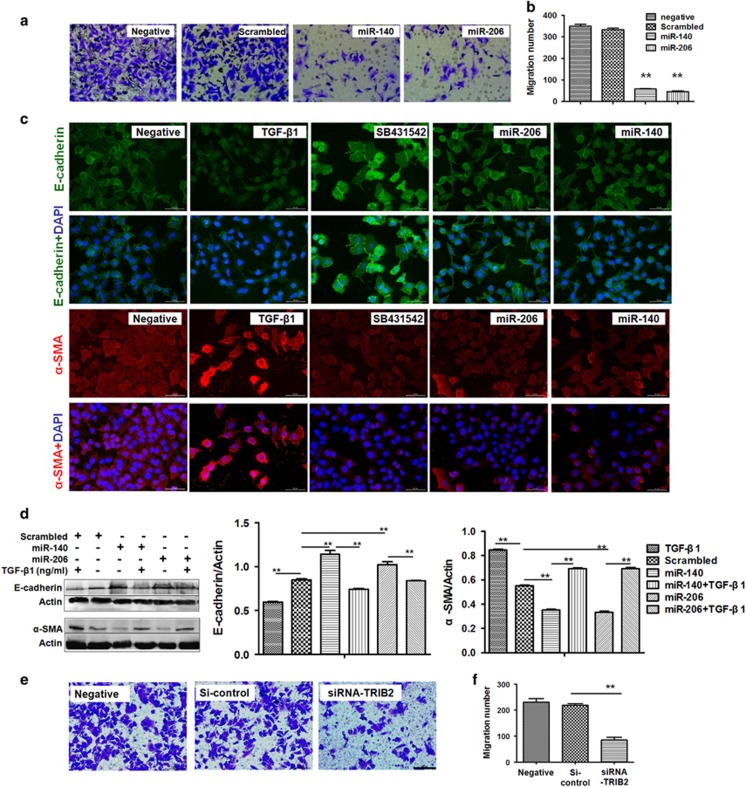
MiR-206 and miR-140 inhibited cell metastasis. (**a** and **b**) Cell metastasis analysis. Fewer A549 cells migrated to the lower chambers in miR-206- or miR-140-treated cultures compared with NC treatment. **P<0.01, miR-206 or miR-140 treatment *versus* scrambled control. (**c**) Immunofluorescence and (**d**) western blot. E-cadherin expression increased, whereas *α*-SMA decreased in miR-206- (or miR-140)-treated A549 cells, similar to data from SB431542 treatment. Relative values for E-cadherin or *α*-SMA *versus* actin are indicated to the right of the gels. **P<0.01, miR-206 treatment *versus* scrambled control. *P*<0.05, miR-140 treatment *versus* scrambled control. MiR-206 or miR-140, cells treated with miR-206 or miR-140 oligos. SiRNA-Smad3 or SiRNA-control, cells treated with small interfering RNA specific to Smad3 or siRNA-control. TGF-*β*1 and SB431542, cells treated with TGF-*β*1 (10 ng/ml) and SB431542 (5 *μ*mol/ml) for 24 h. Bar=50 *μ*M. (**e** and **f**) Cell metastasis analysis. Fewer A549 cells migrated to the lower chambers in siRNA-TRIB2—treated cells compared with siRNA control treatment. **P<0.01 siRNA treatment *versus* siRNA control

MiRNAs have been reported to inhibit tumor metastasis in hepatocellular or ovarian cancer cells.^26, 27^ In our studies to learn how miRNAs function in lung adenocarcinoma metastasis, we found that fewer cells migrated to the lower chamber in miR-206- (or miR-140)-treated cultures compared with scrambled oligo-treated cells (Figure 5a and b), suggesting that miR-206 or miR-140 can inhibit lung adenocarcinoma metastasis. Smad3, a target of miR-206 or miR-140, has a role in tumor cell metastasis and E-cadherin and *α*-SMA^12^ expression, so we measured these proteins in miR-206- (or miR-140)-treated A549 cells. We found that E-cadherin was elevated, and *α*-SMA was decreased in miR-206- (or miR-140)-treated A549 cells, similar to data observed after SB431542 treatment (Figure 5c). Western blot confirmed that miR-206 and miR-140 increased E-cadherin and downregulated *α*-SMA expression in A549 cells (Figure 5d,Supplementary Figure 7). However, using TGF-*β*1 to induce p-Smad3 overexpression enhanced *α*-SMA and downregulated E-cadherin in TGF-*β*1-treated cells, which could be ameliorated in miR-206- (or miR-140)-treated cultures compared with control treatment (Figure 5c and d,Supplementary Figure 7). Because miR-206 and miR-140 also affects Smad3-related TRIB2 expression, we next investigated how TRIB2 contributes to cell migration. Relatively fewer cells migrated to the lower chamber in siRNA (specific to TRIB2)-treated cultures compared with siRNA control-treated cells (Figure 5e and f), supporting that the inhibition to cell migration by miR-206 and miR-140 may also attribute to Smad3-related TRIB2 expression.

**Figure 6:**
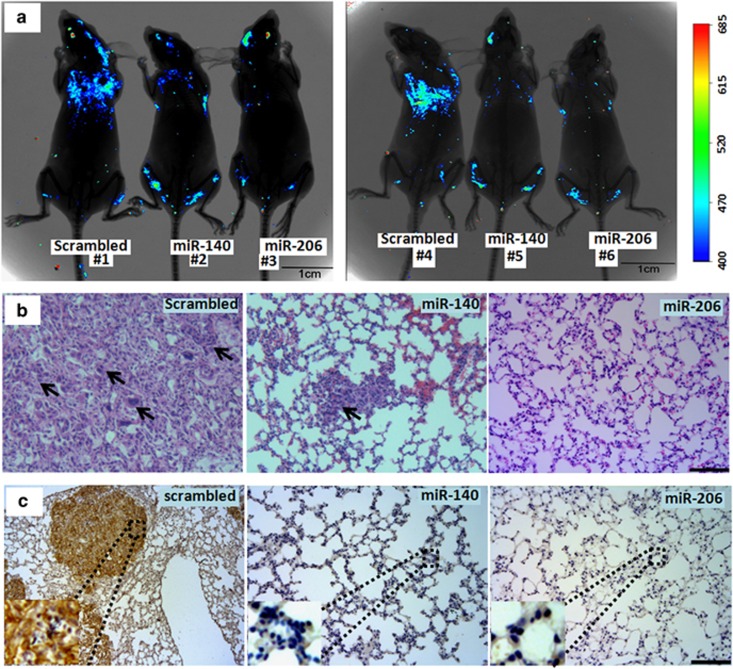
MiRNAs suppressed cell metastasis *in vivo*. (**a**) An experimental metastasis mouse model was injected with control miR-206, miR-140, or scrambled control oligos-treated A549/34 R cells. (**b**) Visualization of the HE-stained lung section. Arrow, the migratory A549 cells. (**c**) Immunohistochemistry was conducted to detect CD44 expression. Brown color indicates the migratory A549 cells. Bar=100 *μ*m

To study the effects of miR-206 and miR-140 on the metastasis of A549 cells *in vivo*, 2 × 10^6^ GFP-positive A549 cells transfected with miRNAs were injected into male nude mice by tail vein. Seven weeks after injection, small animal *in vivo* imaging results showed that fewer GFP-positive A549 cells migrated to the lungs in nude mice treated with miR-206 or miR-140 compared with scrambled control treatment (Figure 6a). In addition, HE staining of lung sections also supported that miR-206 or miR-140 decreased migratory tumors in miRNAs-treated tumors (*n*=3) compared with scrambled control treatment (*n*=3, Figure 6b). As a cell adhesion molecule, human CD44 increases the migratory capacity of various cancers.^28^ We further detected human CD44 expression to analyze the migratory capacity and number of A549 cells in oligo-treated metastatic nodules by using anti-human specific CD44 primary antibody. Interestingly, human CD44 expression decreased in miR-206- (or miR-140)-treated metastatic A549 cell nodules compared with that in scrambled control-treated metastatic nodules (*n*=3, Figure 6c). Therefore, Smad3-related miR-206 and miR-140 could effectively inhibit lung cancer cell metastasis *in vitro* and *in vivo*.

### miR-206 and miR-140 suppressed cell proliferation *in vivo* through TRIB2

**Figure 7:**
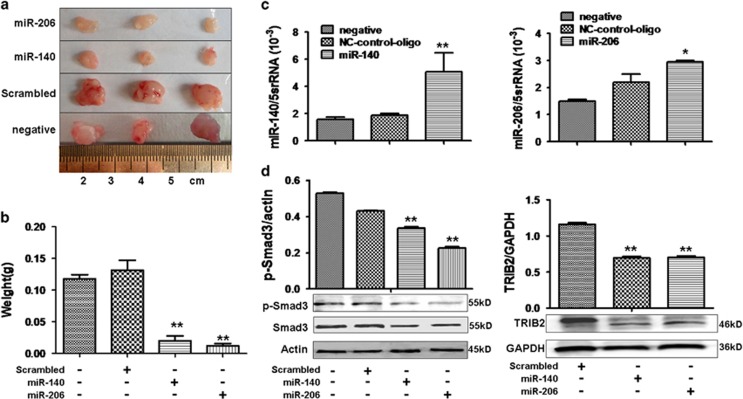
A549 lung cancer xenografts were inhibited by miR-206 and miR-140. (**a**) miR-206 and miR-140 suppresses growth of A549 lung cancer xenografts in BALB/C-nu mice. (**b**) Tumor weight was reduced in miR-206- or miR-140- treated xenografts compared with scrambled-treated controls (**P<0.01, miR-206 or miR-140 treatment *versus* scrambled control, *n*=3). (**c**) Real-time PCR. miR-206 (or miR-140) was higher in miR-206- (or miR-140)-treated xenografts compared with control treatment (*P<0.05, miR-206 *versus* scrambled,**P<0.01, miR-140 *versus* scrambled, *n*=3). (**d**) Western blot indicated reduced expression of p-Smad3 and TRIB2 in miR-206- or miR-140-treated xenografts compared with controls. **P<0.05 miR-206 (or miR-140) treatment *versus* scrambled control. Relative values for p-Smad3/actin or TRIB2/GAPDH are indicated in upper panels. Negative, mock transfections. Scrambled, tumor cells treated with scrambled oligo control RNA. miR-206 or miR-140, tumor cells treated with miR-206 or miR-140 oligos

To evaluate the roles of miR-206 and miR-140 in the regulation of cell proliferation *in vivo*, A549 lung cancer xenografts were established in BALB/C-nu mice. Tumor volumes and weights were smaller in miR-206- (or miR-140)-treated xenografts compared with scrambled-oligo controls (Figure 7a and b). qRT-PCR data show that miR-206 (or miR-140) increased in miR-206- (or miR-140)-treated xenografts compared with control tumors (Figure 7c). P-Smad3 (or Smad3), the target of miR-206 (or miR-140), was lower in tumors treated with miR-206 (or miR-140) compared with control tumors (Figure 7d). TRIB2 expression also decreased in miR-206- (or miR-140)-treated xenografts compared with control treatment (Figure 7d). The suppressive action of miRNA to tumorigenicity may be attributed to downregulation of TRIB2, which was supported by our previous study,^21^ demonstrating that lower levels of TRIB2 lead to inhibiting lung adenocarcinoma cell growth *in vivo*.

### Smad3 and TRIB2 expression in lung adenocarcinoma samples and clinical outcomes

**Figure 8:**
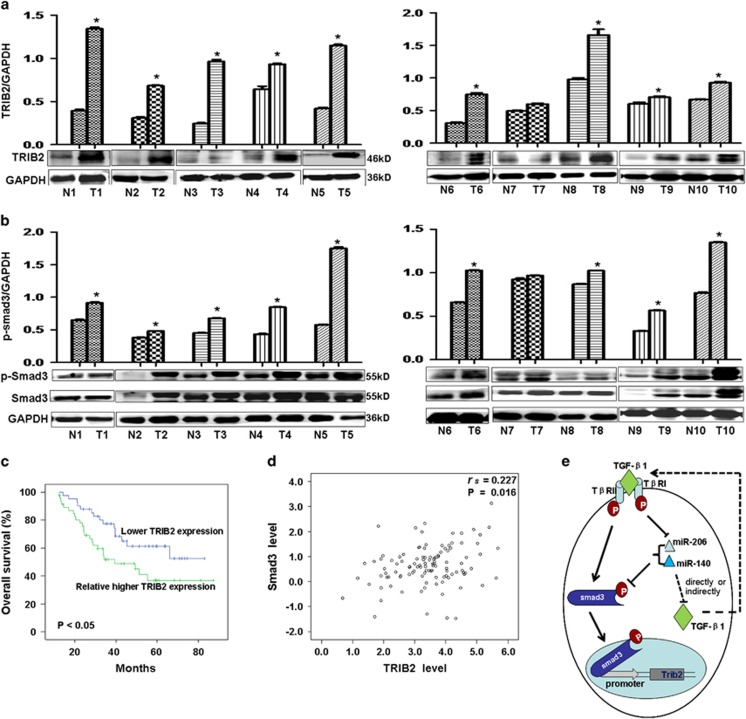
miRNAs, Smad3, and TRIB2 in cancer samples correlated with clinical outcomes. (**a** and **b**) Western blot indicated that expression of p-Smad3 and TRIB2 was relatively higher in lung adenocarcinoma samples (*n*=10) compared with para-carcinomas (*n*=10, **P<0.01 *versus* para-carcinomas). Relative values for p-Smad3/GAPDH or TRIB2/GAPDH appear in the upper panels. (**c**) Kaplan–Meier survival analysis indicated that relatively higher TRIB2 expression is associated with poorer survival in lung cancer patients, *P*<0.05 *versus* lower TRIB2 levels. (**d**) A significantly positive correlation (*r*_s_=0.227, *P*=0.016) was found between Smad3 and TRIB2 in lung cancer patients (*n*=111). (**e**) This study proposed model by which miR-206 (or miR-140) regulating Smad3 and TRIB2 in TGF-*β*1 pathway. p-Smad 3 binds to *trib2* gene promoter to control its expression. miR-206 (or miR-140), negatively regulated by TGF-*β*1, can regulate the levels of Smad 3 in TGF-*β*1 pathway, which may in turn affect TGF-*β*1 levels

We measured Smad3 and TRIB2 expression in lung adenocarcinoma samples. Different to lower levels of miR-206 and miR-140 in adenocarcinoma samples, we noted that p-Smad3, Smad3, and TRIB2 were higher in adenocarcinoma samples compared with para-carcinomas (*n*=10, Figure 8a and b, *P*<0.01), and this was negatively correlated with miR-206 and miR-140 expression, which supporting that the suppressive roles of miR-206 (or miR-140) in regulating the expression of p-Smad3/Smad3 and TRIB2.

Kaplan–Meier survival analysis indicated that patients with greater TRIB2 had a poor survival (Figure 8d). TRIB2 was significantly positively correlated with Smad3 (*n*=111, *r*_s_=0.227, *P*=0.016, Figure 8d), suggesting a positive regulation of Smad3 to TRIB2 promoter activity. These data with the luciferase experiments *in vitro* indicates that Smad3 can increase TRIB2 expression.

## Discussion

MiRNAs are involved in cell proliferation, metastasis, apoptosis, and stress responses^29^ and miR-206 and miR-140 are important suppressors of lung adenocarcinoma cell proliferation and metastasis. We observed there might be a negative feedback loop between miR-206 (or miR-140) and TGF-*β*1 whereby these two miRNAs, downregulated by TGF-*β*1, participated in Smad3-dependent TGF-*β*1 signaling and negatively regulated TGF-*β*1/Smad3 signals in lung adenocarcinoma (Figure 8e). After decreasing p-Smad3 expression, we observed that oncogenic TRIB2 was also regulated by miR-206/miR-140. As tumor suppressors, miR-206 and miR-140 can inhibit lung adenocarcinoma cell metastasis by increasing E-cadherin and decreasing *α*-SMA expression, and suppress lung adenocarcinoma cell growth *in vivo* by decreasing oncogenic TRIB2 promoter activity through Smad3.

MiR-140 was first identified (in chondrocytes) to play a role in cartilage development and homeostasis.^30^ Compared with normal control tissues, miR-140 was downregulated in human ovarian cancer and basal cell carcinoma^31, 32^ and gastric cancer. MiR-140 overexpression inhibited HGC-27 cell viability and colony formation, and resulted in G0/G1 arrest by suppressing SOX4 expression.^33^ In this study, we found that miR-140, as a novel miRNA directly regulating Smad3, acted as a tumor suppressor to inhibit lung adenocarcinoma proliferation and was downregulated in lung adenocarcinoma samples compared with para-carcinomas, a finding supported by Tan *et al.*'s study.^34^ MiR-206 has also been reported to be a tumor suppressor that can block cell proliferation, migration, invasion, and tumorigenesis and induce apoptosis by regulating VEGF expression.^35^ Recently, miR-206 was found to effectively inhibit stemness and metastasis of breast cancer by targeting MKL1/IL11 pathway.^36^ Here we further explored the roles of miR-206 in lung adenocarcinoma and its new target. We found that miR-206 was down-expressed in lung adenocarcinoma samples and that miR-206 and miR-140 can inhibit lung adenocarcinoma cell proliferation *in vitro* and *in vivo* by downregulating new target-Smad3. Moreover, miR-206 and miR-140 can suppress A549 cell metastasis via regulating the expression of E-cadherin and *α*-SMA, a finding supported by Wang *et al.*'s group who reported that increasing miR-206 leads to cell proliferation arrest and weaker lung cancer cell invasiveness.^37^

The TGF-*β*1 signaling pathway is critical to cell differentiation, development, proliferation, and migration.^38^ Constitutive activation of TGF-*β*1 signaling seems to promote tumor progression through tumor-host cell interactions.^39, 40^ Smad3 is a central downstream modulator, which plays important roles in TGF-*β*1/Smad pathway. In this study, we further investigated the roles of miRNAs in lung adenocarcinoma through TGF-*β*1/Smad pathway, and found that miR-206 and miR-140, as signal factors in TGF-*β*1/Smad pathway, could inhibit lung adenocarcinoma cell proliferation and metastasis by downregulating p-Smad3/Smad3 and that this may be related to p-Smad3's promotion of cell growth or invasion^41^ in TGF-*β*1 pathway. It was reported that p-Smad3 binds other Smad proteins into complexes to promote gene expression via regulating Smad3 responsive promoter activity.^22^ Indeed, we constructed a luciferase vector driven by *trib2* promoter to study whether p-Smad3 binds the promoter to activate TRIB2 transcription, and found that the 2.9 kb component of the *trib2* promoter had the greatest activity for driving luciferase expression. Moreover, our results demonstrated that TGF-*β*1 promoted *trib2* promoter activity, which was abolished by miR-206 and miR-140 treatment. By mutating the Smad-binding consensus sequence CAGACA,^23^ we confirmed that p-Smad3 could bind CAGACA to regulate TRIB2 promoter activity.

Tribbles, which are inhibitors of mitosis, regulate cell proliferation, migration, and morphogenesis during development. In mammals, three Tribble homologs exist: TRIB1, TRIB2, and TRIB3, and all are associated with human malignancies.^42, 43, 44^ Several studies indicated that TRIB2 can act as an oncogene involved in a mouse model of AML by inhibiting transcription factor C/EBP*α*.^24, 45^ In previous studies, we reported an oncogenic role of TRIB2 in lung adenocarcinoma, and proved that miR-511 and miR-1297 could suppress A549 cell proliferation *in vitro* and *in vivo* by suppressing TRIB2 and increasing C/EBP*α* expression.^20^ Here we further demonstrated that TRIB2 were higher in adenocarcinoma samples and patients with greater TRIB2 had a poor survival. Downregulation of TRIB2 by miR-206 and miR-140 inhibited A549 cell migration *in vitro* and *in vivo*. TRIB3 can interact with Smad3 to modulate TGF-*β*1–Smad3 signaling and, as such, is important for tumor progression and metastasis.^18^ These data suggest that TGF-*β*1/Smad3 may further affect TRIB2 expression in lung cancer. Indeed, we observed that TRIB2 expression decreased as p-Smad3/Smad3 was downregulated by miRNA or siRNA. Specifically, miR-206 and miR-140 suppressed lung adenocarcinoma proliferation *in vitro* and *in vivo* by decreasing TRIB2 through Smad3 in TGF-*β*1 pathway. Collectively, our results show that miR-206 or miR-140 can suppress lung cancer cell proliferation by reducing oncogenic TRIB2 through Smad3 regulating *trib2* promoter.

During tumor progression, EMT is critical for conversion of early stage tumors into invasive ones, because it promotes tumor cell infiltration into adjacent tissue and the formation of subsequent metastasis.^46^ TGF-*β*1/Smad3 signaling regulates EMT through Smad3-dependent or -independent mechanisms.^47^ During EMT, epithelial markers E-cadherin and zona occludin-1 are downregulated, whereas mesenchymal markers *α*-SMA and fibronectin are upregulated.^48, 49^ In this study, we investigated a miRNA-mediated mechanism of lung cancer cell migration and found that miR-206 and miR-140 could suppress A549 cell metastasis by regulating p-Smad3 and oncogenic TRIB2. Moreover, E-cadherin expression was upregulated, whereas *α*-SMA was downregulated in miR-206- or miR-140-treated cells, suggesting that the mechanism of suppressing EMT by miR-206 and miR-140 might be related to regulating expression of E-cadherin and *α*-SMA.

Thus, we report that miR-206 and miR-140, as tumor suppressors, induce lung adenocarcinoma cell apoptosis and inhibit cell growth by reducing oncogenic *trib2* promoter activity through Smad3 binding CAGACA box and that they suppress lung cancer metastasis by regulating EMT-related factors. Our work offers essential information about novel targets for the development of new therapeutics for treating lung cancers.

## Materials and Methods

### Lung adenocarcinoma samples

Fresh lung adenocarcinoma and para-carcinoma tissues from patients who underwent surgery at YanTaiShan Hospital were obtained after surgery and immediately prepared for pathological diagnosis, western blot or RNA analysis. All experiments were performed in accordance with relevant guidelines of the Medical Ethics Committee of Binzhou Medical University. Before study inclusion, patients provided written informed consent after study procedures were fully explained.

### RT-PCR and real-time PCR

MiRNAs of lung adenocarcinoma cells, tissues, or mice xenografts were isolated by mirVana miRNA Kit (Ambion, Austin, TX, USA) and poly (A) was added using poly(A) polymerase (Ambion). cDNA was synthesized by RT primer 5′-AACATGTACAGTCCATGGATGd(T)30N (A,G,C or T)-3′. Forward primer used to amplify miR-140 was: 5′-CCAGTGGTTTTACCCTATGGTAG-3′, reverse: 5′-AACATGTACAGTCCATGGATG-3′. Forward primer of miR-206: 5′-TGGAATGTAAGGAAGTGTGTGG-3′, reverse: 5′-AACATGTACAGTCCATGGATG-3′. The Quantitect SYBR-Green kit (Qiagen, Valencia, CA, USA) was used to measure miR-140 and miR-206 with an RG3000 system (Corbett Research, Mortlake, Australia) as follows: denaturing at 95 °C for 3 min; 40 cycles of 95 °C for 30 s, 60 °C annealing for 20 s and extension at 72 °C for 20 s. Then, fluorescence was measured (585 nm).

RNAs were isolated with Trizol (Takara, Otsu, Shiga, Japan). cDNAs were synthesized using RT primer (Poly T) and forward primer to amplify Smad3 was: 5′-AGCACACAATAACTTGGACC-3′ reverse: 5′-TAAGACACACTGGAACAGCGGATG-3'. PCR conditions were 30 cycles of denaturation at 94 °C for 45 s, annealing at 50 °C for 45 s and elongation at 72 °C for 45 s, performed in a PCR machine (Eppendorf, Hamburg, Germany).

### *In situ* hybridization

The paraffin sections were baked at 65 °C for 2 h, then were dewaxed with Xylene and dehydrated in alcohol. Endogenous peroxidase activity in tissues was inhibited in 3% hydrogen peroxide solution in methanol for 10 min at room temperature. The sides were digested with protenase K for 30 min at 37 °C. Following prehybridized at 37 °C for 2 h, the sides were hybridized with hsa-miR-206/140-probes (1:50, Exon Biotech Inc., Guangzhou, China) overnight at 37 °C. Then sides were rinsed and blocked at 37 °C for 30 min. The sides were incubated with mouse anti-digoxingenin antibody (1 : 500, Abcam, Cambridge, UK) for 1 h. The Sides were incubated with SABC-POD at 37 °C for 20 min, and then secondary antibody (PV-6000, ZSGB-Bio, Beijing, China) was applied for 30 min at room temperature. 3,3-Diaminobenzidine (DAB, Santa Cruz Biotechnology, Inc., Beijing, China) solution was used for staining. The sections were observed under a microscope (DM6000B, Leica, Dresden, Germany).

### Cell culture and miRNAs transfection

Lung adenocarcinoma cells (A549/LTEP-a-2) and human cervical cancer (HeLa) cells were obtained from Shanghai Institute of Cell Biology, China. Cells were maintained in 1640 medium (Gibco, Grand Island, NY, USA) supplemented with 10% calf serum (Hyclone, Logan, UT, USA), 100 U/ml penicillin and 100 *μ*g/ml streptomycin at 37 °C with 5% CO_2_.

Then, 1 × 10^6^ cells were transfected with 1 *μ*g miRNA (or plasmids) in 2.5 *μ*l of Lipofectamine 2000 (Invitrogen, Carlsbad, CA, USA) according to the manufacturer's instructions. All transfections were carried out in triplicate. Lung adenocarcinoma cells were treated with various concentrations of TGF-*β*1 (5, 10, and 15 ng/ml, Sino Biological Inc., Beijing, China) for 24 h, and the TGF-*β*1 inhibitor, SB431542 (0.5, 1.5, and 5 *μ*mol/ml; Sigma, St Louis, MO, USA) was applied to study the role of TGF-*β*1 and SB431542 in TGF-*β*1/Smad3 signaling.

### Cell proliferation or apoptosis

Cells proliferation was measured with an MTT assay (Sigma). Cells (1 × 10^4^) in each well of 96-well flat bottom microtiter plates were treated with miR-140, miR-206, and control oligos for 48 h. At 4 h before the end of incubation, 10 *μ*l MTT (5 mg/ml) was added into each well. Supernatant was removed and 100 *μ*l DMSO (Sigma) was added and OD was measured (570 nm) using an ELISA reader (Multiskan FC, Thermo Fisher Scientific, Boston, MA, USA).

Apoptosis was measured by flow cytometry (FACS). Briefly, cells (8 × 10^4^) in each well of 12-well flat-bottom microtiter plates were treated with miR-140 and miR-206 for 48 h. Then, cells were dyed with Annexin V-FITC/PI according to the manufacturer's instructions (KeyGEN Biotech. Co. Ltd., Nanjing, China). Finally, Annexin V-FITC/PI positive cells were counted (Beckman Coulter, Inc., Kraemer Boulevard Brea, CA, USA).

### Construction of pcDNA-GFP-Smad-UTR vector

Smad3-3′-UTR was amplified by PCR from human genomic DNA. Forward primer: 5′-TGGAACTCTACTCAACCCATTG-3′ reverse: 5′-TACATACGCCCAAAGCACCT-3′. PCR was carried out with 30 cycles of denaturation at 94 °C for 45 s, annealing at 54 °C for 45 s, and elongation at 72 °C for 60 s, in a PCR machine (Eppendorf). The Smad3-3′-UTR was cloned into a T vector (Takara) to construct the T-Smad3 vector. The Smad3-3′-UTR was then cut from T-Smad3 and inserted downstream of the *GFP* gene in the pcDNA-GFP vector (described previously).^50^

### GFP assays

GFP-positive cells were observed 24 h after transfection. A549 and LTEP-a-2 cells were trypsinized and gently washed with serum-containing medium. Cells were then collected and centrifuged at 400 × *g* for 5 min. Then, GFP-positive cells were counted by FACS (Beckman).

### Western blot

Lung adenocarcinoma or mice xenograft cells were lysed with lysis buffer (Western of Beyotime, Shanghai, China) according to the manufacturer's instruction. Then, 30 *μ*g of protein was loaded into individual lanes and separated via SDS-PAGE. Protein was then transferred to PVDF membranes, which were blocked with 5% non-fat milk in TBST (50 mmol/l Tris-HCl (pH 7.6), 150 mmol/l NaCl, 0.1% Tween-20) for 2 h at room temperature. Membranes were incubated with rabbit anti-human p-Smad3/Smad3/E-cadherin/TRIB2 /C-EBP-*α*/*β* antibody (1:400, Santa Cruz Biotechnology, Inc., Santa Cruz, CA, USA) or *α*-SMA antibody (1:400, Bioworld Technology, Inc., Minneapolis, MN, USA) in TBST at 4 °C overnight. Membranes were washed with TBST three times. HRP-labeled goat anti-rabbit IgG (1:6000, Beijing Zhong Shan-Golden Bridge Technology Co., Ltd., Beijing, China) was added and samples were incubated for 1 h at room temperature. Finally, membranes assessed with ECL (Boster Immunoleader, Wuhan, China). Actin or GAPDH for each sample was used as a control.

### Promoter and luciferase

Different lengths of TRIB2 (NM_021643) promoter elements were amplified by PCR (Primers appear in Supplementary Table 1). Promoters were cloned into the T vector (Takara) to construct T-promoter vectors. Then, promoters were cut from T-promoter vectors by KpnI/SalI, which were inserted before the luciferase sequence of pGL-basic (Promega, Madison,WI, USA) using KpnI/XhoI, constructing promoter-luciferase expression vectors. The mutated promoter-luciferase vector was constructed using a site-directed Gene Mutagenesis Kit (Beyotime). All constructs were confirmed by DNA sequencing.

The Hela cells were treated with both luciferase reporter plasmid and TGF-*β*1 or other factors. After 24 h, cells were collected and luciferase activity was measured with a Dual-Luciferase Reporter Assay according to the manufacturer's instructions (Promega).

### Transwell cell migration assays

Transwell migration assays were performed using Corning Costar Transwell chambers with filter membranes of 8 *μ*m pore size (Sigma). Cells treated with miRNAs or TGF-*β*1 were seeded into the upper chamber (10^4^ cells per well in 100 *μ*l 1640 medium, FBS-free). The lower chamber was filled with 600 *μ*l 1640 medium supplemented with 10% calf serum. After 24 h, the liquid in the upper chamber were removed and the upper surface was carefully washed with PBS three times. In the upper chamber, −20 °C methanol was added for 10 min and samples were washed with PBS twice. Then, lower chamber cells were stained with 1% crystal violet (Sigma) in 2% ethanol for 20 min. Excess crystal violet was removed by quickly merging the insert in ddH_2_O for 3–4 s. Lower chamber cells were counted under a microscope (DM6000B, Leica). Each migration condition was tested three times.

### Tail vein injection and migratory cell detection

GFP-positive A549 cells transfected with miRNAs or controls were collected from petri dish in 100 *μ*l normal saline at 1.5 × 10^6^ cells. Subsequently, these cells were injected into the tail veins of nude mice (5 weeks old). Seven weeks later, the migration of GFP-positive cells were observed using a Small Animal *In vivo* FX Pro (FX PRO, Bruker, Sweden).

Then the lungs were dissected, and hematoxylin–eosin staining (HE) and immunohistochemistry was performed as previously described.^51^ The sections were incubated overnight with mouse anti-human CD44 primary antibodies (1:50, Boster Biological Technology Co., Wuhan, China) at 4 °C, and secondary antibody (PV-6000) and DAB solution were used for detection. The sections were observed under a microscope ((DM6000B, Leica).

### Immunofluorescent analysis

Cells were fixed with 4% paraformaldehyde in PBS, permeabilized with 0.5% Triton X-100 in PBS, and incubated with rabbit anti human E-cadherin (1:50; Santa Cruz Biotechnology), *α*-SMA (1:100; Bioworld Technology) at 4 °C overnight. Then, cells were incubated with Alexa Fluor 488 donkey anti-rabbit IgG (H+L) and Alexa Fluor 594 donkey anti-mouse IgG (H+L) (Molecular probes, Eugene, OR, USA) at 37 °C for 1 h. Fluorescent images were captured under a microscope (DM6000B, Leica).

### A549 lung adenocarcinoma cell xenografts

Briefly, after treatment with miR-140 or miR-206 for 48 h, A549 cells were cultured, collected, washed, and resuspended in culture medium (~2 × 10^7^/ml) and injected into the lower back of 6–8-week old female BALB/C-nu mice (nude mice, HFK Bio-Technology, Beijing, China). Once mice developed palpable tumors, tumor volume was measured with calipers daily. All mice were killed after 4 weeks and tumors were collected. All animal experiments were approved by the Committee on the Ethics of Animal Experiments of Binzhou Medical University.

### Statistics

SPSS Statistics Client 22 (IBM) software was used to analyze the significance of all results. Group means comparisons were calculated using an unpaired, two-sided, Student's *t*-test. ANOVA was applied to compare different groups with respect to continuous variables. Array data of TRIB2 and Smad3 were downloaded from data link Data Link(s): http://www.ncbi.nlm.nih.gov/geo/query/acc.cgi?acc=GSE3141. Overall survival was determined using Kaplan–Meier survival analysis. Correlations were calculated with a Spearman rank test. *P*-values <0.05 were considered statistically significant differences.
