# Supplementary material for: Smad3-related miRNAs regulated oncogenic TRIB2 promoter activity to effectively suppress lung adenocarcinoma growth
Source: Cell Death Dis. 2016 Dec 22;7(12):e2528–. doi: 10.1038/cddis.2016.432 (PMC5260984; doi:10.1038/cddis.2016.432)

Supplemental Table 1. The primers used to amplify trib2 promoter.

Primers	5'— 3'	Promoter Length (bp)
F1	GCAAGACATTGTGCCAGATGCT	2908 (F1,R1)
F2	CGTGTCACTTCCCAATGTCAGC	2412 (F2,R1)
F3	TGACTGTTGTCCCAAGTGGA	1241 (F3,R1)
R1	TTTCCACGGAGCCTCCGC	---

Supplemental Figure 1. miR-206 and MiR-140 induced LTEP-a-2 cell apoptosis

(a) miR-206 inducing cell apoptosis. (b) miR-140 inducing cell apoptosis. Apoptotic cells are shown in the upper left, upper right, and lower right quadrants of each panel. More apoptotic cells were found in the miR-206- or miR-140-treated cells compared with the scrambled oligo control-treated cells after Annexin V-FITC/PI staining. (c) MTT assay. OD was more in miR-206 inhibitor (ASO-206)- ($P < 0.01$) or miR-140 inhibitor (ASO-140)- treated A549 cells ($P < 0.01$) compared with miRNA- or scrambled oligo-treated cultures. (d) Colony formation assay. A549 cells, treated with miR-206, miR-140 and controls, were seeded on six-well plates at a density of 500 cells/well. After 12 days, the colonies were fixed with methanol and stained with 0.1% crystal violet (Sigma, St. Louis, MO). ASO-206- or ASO-140 increased colony formation compared with the miRNA or scrambled oligo control treatment. Scrambled, cells treated scrambled control RNA. MiR-206 or miR-140, cells treated with miR-206 or miR-140 oligos. ASO-206 or ASO-140, cells treated with antisense RNA specific to miR-206 or miR-140.

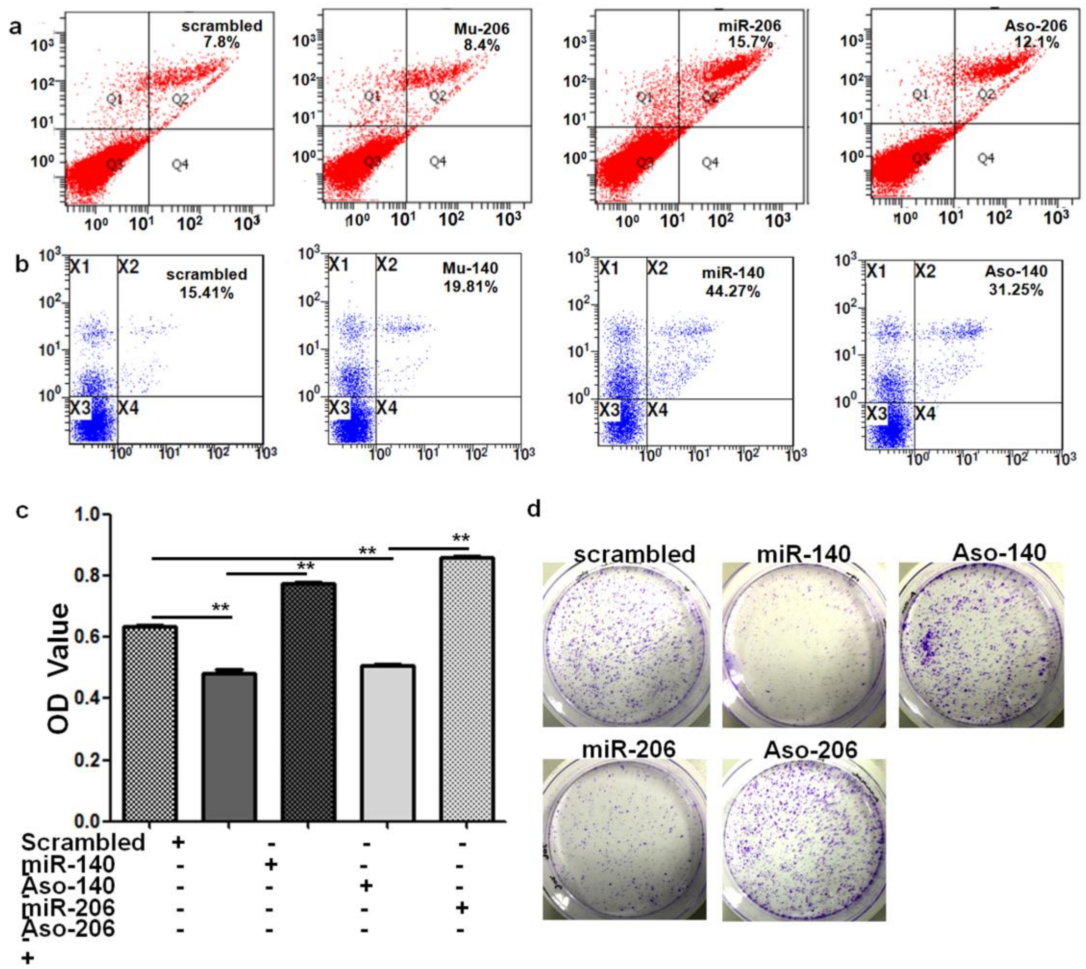

Supplemental Figure 2. Smad3 expression was regulated by miRNAs in LTEP-a-2 cells.

(a) Fluorescence observation. Scale bar = 100 μM. (b) FACS analysis. MiR-206 and miR-140 treatment reduced the number of GFP positive cells and intensity of GFP fluorescence significantly compared with control oligos treatment. (c) Smad3 and p-Smad3 protein (55kD) expression were detected by western blot. The results showed that p-Smad3 and Smad3 expression was inhibited by miR-140 or miR-206 treatment compared with control treatment. The Smad3/actin (or p-Smad3/actin) was shown on the right of the gel. ** p<0.01. miRNA treatment *vs*. control treatment. scrambled, cells treated scrambled control RNA. MiR-206 or miR-140, cells treated with miR-206 or miR-140 oligos. Mu-206 or Mu-140, cells treated with mutation sequence of miR-206 or miR-140. SiRNA-smad3, small interfering RNA specific to knock down Smad3 expression.

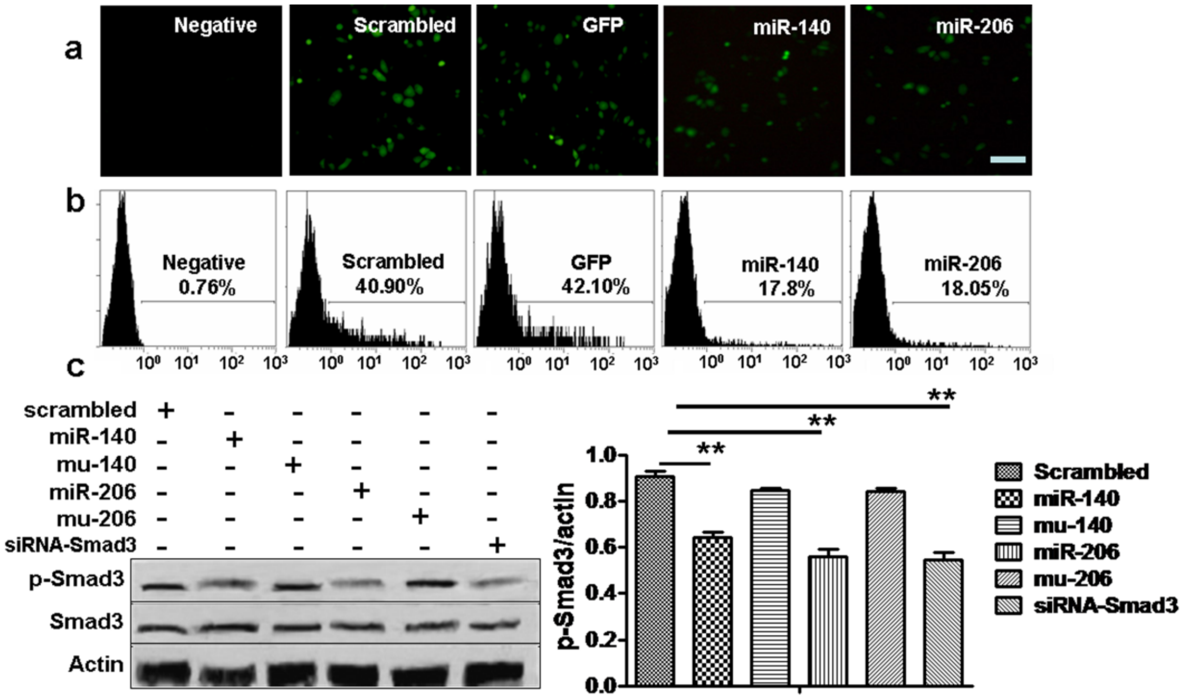

Supplemental Figure 3. The effect of TGF-β1 on miR-140 or miR-206 expression in LTEP-a-2 cells. (a) miR-140, (b) miR-206. The levels of miR-140 (A) and miR-206 were obviously decreased in TGF-β1-treated compared with untreated LTEP-a-2 cells. **p<0.01. n = 3 replicates.

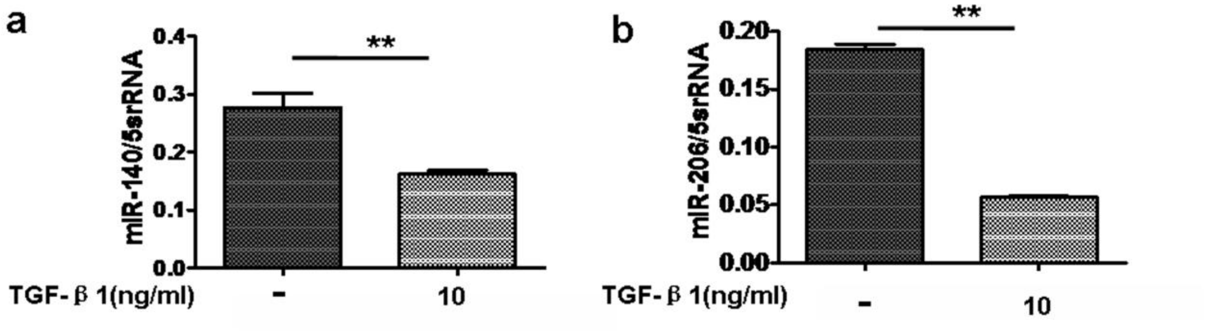

Supplemental Figure 4. TRIB2 expression in LTEP-a-2. TRIB2 expression (46kD) was decreased obviously in miR-140- or miR-206-treated cells. Relative values for TRIB2 vs Actin are indicated to the right of the gel. **p<0.01. *vs.* negative control. n = 3 replicates. MiR-206 or miR-140, cells treated with miR-206 or miR-140 oligos. Mu-206 or Mu-140, cells treated with mutation sequence of miR-206 or miR-140. SiRNA-Smad3, cells treated with small interfering RNA specific to Smad3. The sense sequence of siRNA-Smad3, 5'-CACAUAAUAACUUGGACCUU -3'.

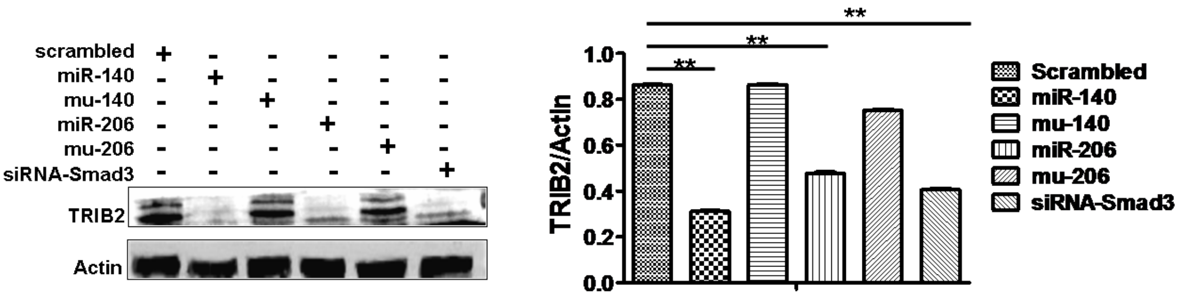

Supplemental Figure 5. Mouse TRIB2 promoter activity analysis. TGF-β1 treatment increased mouse trib2 promoter activity, but SB431542 blocked TGF-β1-mediated mouse trib2 promoter activity. **p<0.01 vs. TGF-β1 treatment.

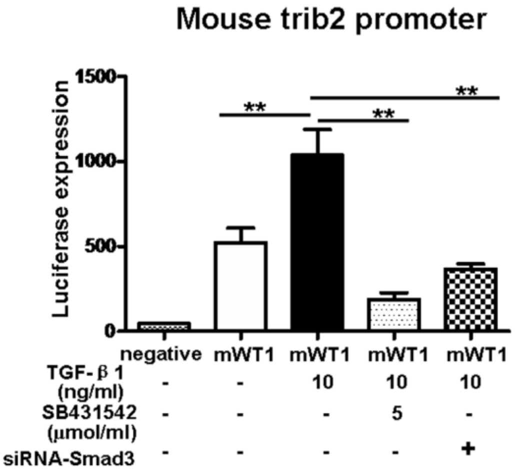

Supplemental Figure 6. Mutant of "CAGACA" box. The "CAGACA" box was missed in mutation promoter compared with wide type promoter.

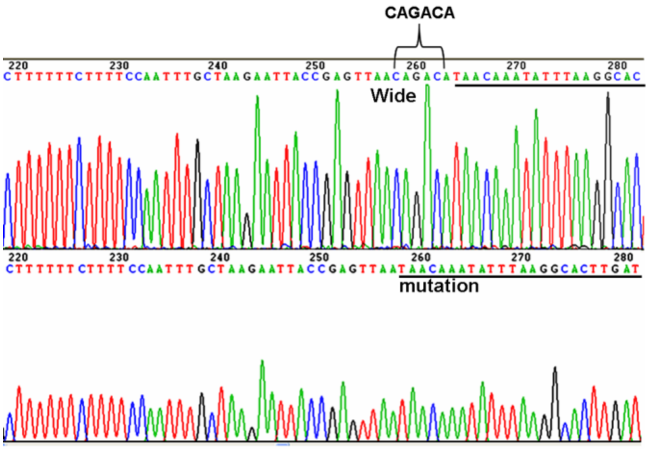

Supplemental Figure 7. The expression of E-cadherin and α-SMA in LTEP-a-2 cells. Western Blot showed that the expression of E-cadherin (130kD) was increased, while α-SMA (45kD) level was reduced in miR-140- (or miR-206-) treated LTEP-a-2 cells. Relative values for E-cadherin or α-SMA vs Actin are indicated to the right of the gel. **p<0.01. Scrambled, cells treated scrambled control RNA. MiR-206 or miR-140, cells treated with miR-206 or miR-140 oligos. SiRNA-Smad3 or SiRNA-control, cells treated with small interfering RNA specific to Smad3 or siRNA-control. TGF-β1, 10ng/ml TGF-β1 treatment. SB431542, 5 μmol/ml SB431542 treatment.

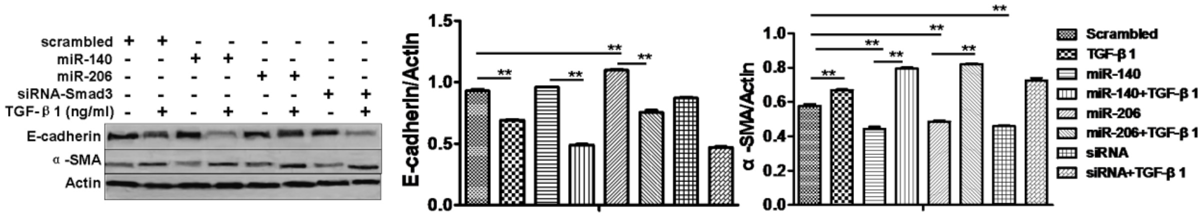

Supplement: Supplementary Information [file cddis2016432x1.pdf]
